# The ethical decisions UK doctors make regarding advanced cancer patients at the end of life - the perceived (in) appropriateness of anticoagulation for venous thromboembolism: A qualitative study

**DOI:** 10.1186/1472-6939-13-22

**Published:** 2012-09-04

**Authors:** Laura Sheard, Hayley Prout, Dawn Dowding, Simon Noble, Ian Watt, Anthony Maraveyas, Miriam Johnson

**Affiliations:** 1 Area 2, Health Sciences, Seebohm Rowntree Building, University of York, York, YO10 5DD, England; 2 Department of Primary Care and Public Health, School of Medicine, Cardiff University, Cardiff, Wales; 3 School of Healthcare, University of Leeds, Leeds, England; 4 Department of Palliative Medicine, Cardiff University, Cardiff, Wales; 5 Health Sciences, University of York, York, England; 6 Department of Academic Oncology, Queens Centre for Oncology and Haematology, Castle Hill Hospital, Hull, England; 7 Hull York Medical School, University of Hull, Hull, England

**Keywords:** Venous thromboembolism, Heparin, Low-molecular-weight, Palliative care, Qualitative research, Ethics, Medical

## Abstract

**Background:**

Cancer patients are at risk of developing blood clots in their veins - venous thromboembolism (VTE) - which often takes the form of a pulmonary embolism or deep vein thrombosis. The risk increases with advanced disease. Evidence based treatment is low molecular weight heparin (LMWH) by daily subcutaneous injection. The aim of this research is to explore the barriers for doctors in the UK when diagnosing and treating advanced cancer patients with VTE.

**Method:**

Qualitative, in-depth interview study with 45 doctors (30 across Yorkshire, England and 15 across South Wales). Doctors were from three specialties: oncology, palliative medicine and general practice, with a mixture of senior and junior staff. Framework analysis was used.

**Results:**

Doctors opinions as to whether LMWH treatment was ethically appropriate for patients who were symptomatic from VTE but at end of life existed on a shifting continuum, largely influenced by patient prognosis. A lack of immediate benefit coupled with the discomfort of a daily injection had influenced some doctors not to prescribe LMWH. The point at which LMWH injections should be stopped in patients at the end of life was ambiguous**.** Some perceived ‘overcaution’ in their own and other clinicians’ treatment of patients**.** Viewpoints were divergent on whether dying of a PE was considered a “good way to go”. The interventionalism and ethos of palliative medicine was discussed.

**Conclusions:**

Decisions are difficult for doctors to make regarding LMWH treatment for advanced cancer patients with VTE. Treatment for this patient group is bounded to the doctors own moral and ethical frameworks.

## Background

People living with cancer often develop blood clots in their veins - venous thromboembolism (VTE) and have a six-fold risk of developing VTE compared to those without cancer [1] The risk increases as the cancer progresses; one survey demonstrated evidence of deep vein thrombosis (DVT) in over 50% of admissions to a specialist palliative care unit [2]. VTE most commonly takes the form of a pulmonary embolism (PE) or DVT. A PE is an obstruction of the pulmonary artery (or branch of it) leading to the lung, by a blood clot. DVT is usually a blood clot in the veins of the lower leg or thigh. A range of symptoms may be caused by a PE from none at all, to severe breathlessness or death, and a DVT may be asymptomatic, or can cause distressing lower limb pain and swelling [3]. A DVT can precipitate a PE, whereby the blood clot travels from the leg to the lungs.

Typical treatment for VTE is anticoagulation therapy (“blood thinning”). Recommended, evidence based treatment to prevent recurrence or extension of VTE for patients with cancer is low molecular weight heparin (LMWH) by daily subcutaneous injection for three to six months. This is preferential to oral warfarin which is the most common treatment for a blood clot in people without cancer. Warfarin is less effective in preventing extension or recurrence of a clot than LMWH and may also carry a higher risk of bleeding complications in cancer patients than LMWH, especially for those undergoing chemotherapy, those at risk of intracranial or metastatic bleeding, and those with advanced disease. UK registry [4] data showed that long term LMWH was not routinely prescribed by clinicians for cancer patients. We conducted a study to explore the barriers to recommended practice in this clinical area.

Management decisions in those with advanced disease but who are not imminently dying can be difficult [5]. Some areas of palliative medicine may be viewed as a particularly ethically fraught when it comes to medical decision making, especially where prognostication is difficult. Recent commentators have discussed the limitations of end of life treatment [6], the end of life decision making process for dementia patients [7] and the quality of ethical guidelines at the end of life [8]. The four prima facie principles of medical ethics – autonomy, beneficence, non-maleficence & justice [9] – allow us a lens through which to view decisions made in this area of medicine, VTE treatment in advanced cancer patients. Decisions in the area of VTE management may represent a tension between the principles of beneficence (do good) and non-maleficence (minimise harm). In this paper we discuss a subset of the findings from a study conducted to explore the barriers to recommended practice for patients with VTE and cancer. The theme of ‘appropriateness’ was identified by doctors who were interviewed during the study as a major part of their decision-making process when managing people with VTE and advanced cancer.

## Method

The wider study on which this paper is based was conducted in two phases. In phase one, 46 doctors were recruited and took part in a ‘think aloud’ exercise which aimed to understand how doctors make decisions regarding advanced cancer patients with VTE. In phase two, 45 doctors participated in an in depth interview to explore the barriers present for doctors when diagnosing and treating this patient group. This paper focuses on the theme of ‘appropriateness’ which arose from the phase two interview data.

NHS Research Ethics approval was obtained in March 2010 and Research Governance approval from 7 NHS Trusts and 3 Welsh Health Boards was granted between July and September 2010.

### Participants

In depth interviews were conducted with 45 UK doctors (30 across Yorkshire, England and 15 in the South of Wales). Fieldwork took place between September 2010 and January 2011. For phase one of the study, doctors at consultant and medical director level were identified by registers in the public domain: hospital websites for oncologists, the current Hospice Directory for palliative medicine clinicians and Primary Care Trust websites for GPs or the websites of individual GP practices. Doctors at SpR or ST grade were identified through Deanery lists. Potential participants were invited by a letter on behalf of the research group, sent by mail or email. Doctors who had taken part in phase one were e-mailed and asked if they would be interested in participating in stage two (on which this paper is based). Thirty two out of the 45 participants were purposively sampled and then recruited in this manner. The majority of the rest were recruited by snowball sampling, seeking to maximize variation in the sample.

The doctors selected for this study were purposively sampled on their occupation specialty and grade as these are the pivotal variables for the wider study. This consisted of: oncologists (15 in England, 5 in Wales), palliative medicine doctors (10 in England, 5 in Wales) and GPs (5 in each site). Oncologists were recruited from two large teaching hospitals, two oncology hospitals and two district general hospitals. Palliative medicine doctors worked in some of the hospitals mentioned above and a wide range of hospices (some participants had a dual hospital/hospice role). GPs worked across North Yorkshire, South Glamorgan, Mid Glamorgan & Gwent. Variation within specialties was maximized by ensuring a mixture of senior and junior staff. This allowed for opinion based on the differing experiences of doctors at different stages in their career and training to be accessed. Participants ranged in age from 28 to 58 years with 26 women and 19 men. They practised across a geographically broad area in both sites, covering all four ridings of Yorkshire and a large section of South Wales.

### Data collection

The initial topic guide focussed on the barriers and facilitators to practice around VTE and cancer. Most of the interviews began with the broad question “can you tell me what you think the barriers are to the diagnosis and treatment of cancer patients with VTE?” Questioning about the barriers to practice for VTE in cancer patients centred on: anticoagulation; diagnosis & treatment of VTE; logistical, clinical, institutional or attitudinal issues; positive facilitators to good practice. Questioning was adaptive to the responses of the participants.

The topic guide was restructured and amended throughout the fieldwork as new themes emerged. Interviews were largely conducted at the participants’ place of work although a minority of participants were interviewed in their home. All participants provided written, informed consent prior to taking part in the study. Interviews lasted between 20 and 70 min. The interview was taped with a digital recorder and the digital file was transcribed.

### Analysis

We used Framework Analysis [10] whilst maintaining the notion that analysis is “constantly iterative” [11]. Framework Analysis begins from the aims and objectives of the study yet is inherently grounded in the opinions and experiences of the population studied. DD and LS independently scrutinised the same six transcripts, which were selected for maximum variation. The initial coding framework was devised after comparison of resulting draft frameworks, discussion and subsequent revision. LS and HP coded all the interviews and reached full agreement on coding after four interviews, thereafter coding independently all subsequent interviews. All data were inputted into Atlas.ti which allowed us to effectively sort the data. LS conducted the final two stages of analysis: charting, mapping and interpretation. This creative process involves defining concepts and mapping polarities to identify the range and extent of a phenomenon. Themes were written up describing the similarity and variation between participant’s experiences.

The themes reported in this paper are only one portion of the wider study. Other pertinent themes were: logistical & organisational issues, VTE risk assessment tools, patient & disease specific factors and knowledge, evidence & experience.

## Results

This paper emphasises the agenda of the doctors interviewed - treatment decisions for them were less concerned with the choice between LMWH and warfarin (as most were found to be using LMWH) and more concerned with whether treatment *itself* could be considered appropriate for patients with advanced cancer. Doctors held widely discrepant opinions as to whether LMWH injections should be administered to patients who were symptomatic from VTE but only had weeks or days to live.

### Continuum of appropriateness

The opinions of the participants in relation to what they considered appropriate for advanced cancer patients with VTE existed on a continuum. Some doctors had strong viewpoints that patients should not receive anticoagulation (or be investigated for VTE) if they were believed to be in the last few weeks of life whereas doctors who were at the opposite end of the continuum believed that it was the right of the patient to be treated and rejected notions they perceived as paternalistic. Few participants believed that patients at the end of life should *always* be treated for VTE as the doctors decisions often continually shifted based on context and – most importantly – patient wishes and prognosis.

Many doctors wondered if it was “fair” to give a patient a daily subcutaneous injection of LMWH in the last few weeks of life. Doing so was said to worsen the quality of life in the short time which the patient had left due to the assumed pain and subsequent bruising by administering the injection into a patient’s abdomen. This fed into debates about whether patient’s lives were being prolonged unnecessarily as a result of anticoagulation. A phrase used by a few participants was “doing more harm than good” with a sometimes associated sense of futility. A senior GP in England outlines this:

"If you’ve got terminal cancer, something is going to kill you and for the quality of life of your last few days does it matter if you live several hours less because you’ve got a thromboembolism than having your last few days dreadful because people have been sticking needles in you on a regular basis (England, ID 14)."

In the middle of the continuum, a range of factors were said to influence a doctors decision as to whether they considered it appropriate to treat with LMWH injections. The pivotal variable appeared to be the prognosis of the patient with treatment said to be largely unbeneficial at the end of life due to the fact that in the short time left, patient’s symptoms may not be ameliorated by LMWH injections. A few participants explained how there was a lack of immediate benefit from a LMWH injection, especially for a DVT. This lack of immediate benefit contrasts with some other medical situations in which a patient would receive an injection and sometimes influenced clinicians, as this palliative medicine consultant in England discusses:

"Normally in a hospice context, when you give an injection it’s for pain relief or nausea and you’re expecting an immediate return on what you do and so you off-set the discomfort for the patient and the staff member of giving and receiving the injection by the immediate reward of symptom relief. With low molecular weight heparin for venous thromboembolism, some of the archaic attitude is, “well either it’s happened anyway and if it happens again, it’s a nice way to go”, compounded by the fact that the patient isn’t receiving any immediate comfort from the injection and, [the nurses say] “oh look very bruised” (England, ID 3)"

Whereas treatment for a DVT may see no immediate benefit, LMWH injections can relieve breathlessness in people with PE and was seen as a situation in which some doctors considered it appropriate for dying patients. A palliative medicine consultant in Wales explains this:

"If you’ve got sudden central crushing chest pain and you can’t breathe and you’re scared stupid and your family, you would want to be admitted [to hospital] and I think that’s one of the things that probably directs me that it’s an acute presentation and you’re treating people, not just because you thinking you’re adding days to their life but also it hurts a lot and for lots of people you would, if you identify and treat them then it is symptom management as well as time and also in terms of quality because if you’ve had loads of thrombus showers, then in functional terms, even if you don’t die today, you will have functional impairment because of breathlessness because of your post thrombotic complications in your lungs really, so it’s symptom control. (Wales, ID 13)"

One of the main difficulties in following the debate is that, the term ‘end of life’ (sometimes used interchangeably with the term ‘dying’) was clearly understood in a variety of ways by the participants. Some used the term interchangeably with “dying now”. However, most described end of life as the last hours or few days but a minority talked about it as a much longer timescale of at least weeks. This has implications for the stage at which doctors would perceive LMWH injections as unsuitable as the defining point of ‘end of life’ was so variable and it was therefore difficult to pinpoint a general consensus. The point at which – or even whether - LMWH injections should be stopped when someone was coming towards the end of life was perceived as a difficult judgement to make. This was wrapped in the tension as to whether dying patients should be “interfered” with and even whether palliative medicine intervenes too much in this instance, as this palliative medicine consultant in Wales states:

"I think doing nothing a lot of the time is good practice and you shouldn’t be meddling just because there is a treatment available…If they’re becoming comatosed, if they’re not drinking, if they’re not taking oral medication, then what are we doing jabbing them? You’ve got to be thinking about the time limit of it [life expectancy] and is it helpful to do that? (Wales, ID 4)"

Some participants questioned whether they were too overcautious in relation to making decisions about treatment. Over-caution was a contradictory concept: some perceived themselves – and others – as being too cautious to stop the injections (usually for fear of the patient becoming breathless) whilst others would have already stopped the injections to spare the patient what they felt was unnecessary intervention. A palliative medicine consultant in England outlined both sides of this contradiction:

"I think because we look after people who have got quite advanced disease and you know their prognosis is short and what you don’t want to do is make anything worse because you would just feel awful…So for example if you think someone may or may not have a DVT but they are otherwise quite poorly but comfortable, if they are not getting a lot of symptoms about that then it’s being overcautious about do I interfere with the rest of the quality of their life when they are not that symptomatic. And I think you can forget that they might just go and have a big PE and then they would be far more symptomatic. And then you think well if they are going to die relatively shortly, as long as they have a really big PE and go really quickly then it is not going to be too awful a thing. Your mind goes round all these things and then you think, well what if they just have a moderate sized PE and they are really breathless but don’t die (England, ID 8)"

A senior palliative medicine doctor also in England describes how she felt other staff were being too over-cautious when it came to stopping the injections:

"I think it’s easy to start something [LMWH injections] and then it become inappropriate and doesn’t get stopped… Lots of patients come to their last few days of life who are still on it and there can be quite a lot of anxiety about stopping it. You know, junior doctors agonise over stopping it, even if the patient is on the Care Pathway in the last 48 h, why are their agonising? I think the perception of the risk of stopping it is amplified to what it should be (England, ID 6)"

Ethical decisions surrounding investigation largely mirrored those of treatment. It was considered inappropriate by a minority to subject the patient to what was said to be the distressing journey to hospital (and possible inpatient stay in a setting ill-equipped to manage complex symptoms in people with advanced cancer) if they were at the end of life. If the doctor had already decided not to treat the patient then there was said to be very little point in confirming or excluding a PE or DVT diagnostically. However, it did appear that in these unwell patients, logistical difficulties with regard to accessing diagnostic services had a more disproportionate effect on the decision not to investigate and treat than if the patient had a better performance status.

### A good way to die?

Debate was present about whether dying from a PE could be considered a good way to die – “a good way to go” and opinions were put forward which both supported and opposed this idea. Some of these opinions have already been reflected in the quotations outlined previously, as discussion about whether a PE was a good way to die was prevalent throughout the interviews. A previous qualitative study suggested that doctors believed dying from a PE to be a quick, painless way to die [12] but evidence [13] suggests death is rarely instantaneous and can be distressing and painful. Many of the doctors working in hospices described how the idea of a PE being a good way to die was often prevalent amongst nursing staff, as this palliative medicine registrar in England explains:

"I suppose in their [nurses] mind they see a PE as a sudden event which takes them [patients] away and that is a good thing. Maybe it is a lack of experience of seeing that actually PEs aren’t always fatal and they can actually be debilitating rather than killing somebody. I suppose it is a paternalistic approach of wanting to care for that patient and protect them from harm and hoping that is the way they are going to go but it is not under our control to do that. So I think it is a slightly misheld belief based on a little bit of experience but not enough experience. It’s transferring their views onto that patient…I think it is just that they care so strongly and they get so involved with our patients and quite rightly you get to know them so well that you get protective over them and they don’t want us to stab them with needles which will make them bleed if actually in their mind they might die peacefully in their sleep. But unfortunately it is not always that way (England, ID 2)"

When it came to doctors’ own opinions on this matter, an oncology consultant and a palliative medicine consultant - both from Wales - outlined their contrasting stances respectively:

"Do you want to be treating people with VTE and PE and do you want to be running around and looking for it in cancer patients with very bad prognosis and very little time to live? No. Should I be giving them all low molecular weight heparin injections into their stomach when they are dying? No, I don’t think so. Maybe that is why this VTE business was [previously] not looked for in patients with advanced cancer because we are thinking that their prognosis is quite limited and some would argue that is a reasonable way to go. You have cancer and you are in a lot of pain and suffering and all this and then if it is the PE that is going to do it in the end, so what? Are we harming the patient? I don’t think we are harming the patient (Oncology consultant, Wales, ID 7)"

"I’m actively looking to treat anybody who has a PE or DVT because obviously my perspective is that I see people with either horribly unmanaged DVTs or who become very, very breathless with what I presume is a PE and I don’t see that as a good way to die, so I’m very hot on wanting to pick them up, diagnose them and I know I will be picking up and diagnosing only a proportion of what’s probably there but I’m very keen to encourage patients to have the treatment (Palliative medicine consultant, Wales, ID15)"

### Hospice ethos

Debate existed as to whether patients should just be cared for (“tucked up”) at the end of life and whether investigation itself represents an instance of over-medicalisation. With regards to treatment as well as investigation, doctors who worked in hospices often commented on the medical culture of the hospice they worked in. In some more traditional hospices, VTE was not a common clinical consideration and not actively investigated or treated in keeping with the ethos that dying patients should have the least intervention possible, as this palliative medicine registrar in England describes:

"The most recent hospice that I’ve worked in was very traditional, old style hospice and everything you did got challenged. I think they very much felt that we should almost be tucking the patients up and letting them die, you know, starting the syringe driver, “we’re not doing anything more than that.” Anything more than that they felt was cruel to the patient and actually doctors just taking over and doing things that they wanted to do rather than in the best interests of the patient and they felt like that quite strongly. So to get a scan [at the hospital] for a patient there, they really would have to justify it with everyone. Whereas if I’d worked in other places they would be, “fine we’ll phone up and we’ll organise transport for you and we’ll sort this bit out” (England, ID 1)"

A non-interventionist ethos had angered and frustrated a few participants. One palliative medicine registrar made the comparison between how pneumonia would not be ignored, whereas VTE sometimes is.

## Discussion

This paper has explored the ethical decisions which doctors face for advanced cancer patients with VTE, at the end of life. Whether an action could be considered “appropriate” for this group of patients was a major part of doctors’ decision making strategies. However, defining what is appropriate and what is not seems to be intrinsically related to the individual viewpoints of different doctors, which can be demonstrated by the divergent viewpoints which were held for the same issue. This can clearly be seen when participants discussed the dilemma as to whether dying of a PE is considered a good or bad way to die with contradicting personal opinions of two doctors, both at consultant level, with the same need to maximise quality of life in a short time frame used to support their opposite conclusions. Inherent in the views of those opposed to LMWH for patients at the end of life may be an implicit paternalism given that patient choice is more firmly aligned with the viewpoint of offering LMWH in order to prevent or control the symptoms of a fatal PE. This has direct implications for patient care as two similar patients seen by different doctors may be subject to polarised clinical opinions on administering LMWH, which potentially entails consequences for patient choice and autonomy, controlling symptoms and also life expectancy.

The majority of doctor’s decision making strategies are guided by individual patient context and can be placed on a shifting continuum that pays attention to the complex benefits and burdens of giving treatment for patients near the end of life. General Medical Council guidelines on treatment towards the end of life state:

"…It may be of no overall benefit to provide potentially life-prolonging but burdensome treatment in the last days of a patient’s life when the focus of care is changing from active treatment to managing the patient’s symptoms and keeping them comfortable [14]"

But this obfuscates the fact that LMWH for some patients may be partly or wholly for the purposes of symptom management. This was most often the case to prevent severe breathlessness becoming worse by preventing further PE, thus allowing the symptom to settle. But it was coupled with the burden that an immediate return – within hours - may not be seen with daily injections. This represents an ethical dilemma in itself and it is here where we can see the tension between the principles of beneficence (do good) and non-maleficence (minimise harm). Some doctors may lean towards beneficence upon witnessing patients becoming breathless. However, there is anecdotal concern that a LMWH injection is too invasive and distressing for patients with advanced cancer, both due to the pain of the injection and also the subsequent bruising, especially in the last few days of life. This may sway some doctors towards the principle of non-maleficence based on the potential distress of a subcutaneous injection. However, in a study which looked at treatment injections, a daily LMWH injection was found to be acceptable to patients with advanced cancer [15]. Given this evidence, it could be argued that clinicians who withhold LMWH injections because they do not want to submit the patient to the perceived discomfort of the physical act of the injection are making a moral decision which may run contrary to that of the patient’s own choice. However, this argument has to be put into context of clinicians witnessing patients in distressing circumstances and compassionately not wanting to inflict additional burden on their patient when the immediate cessation of symptoms may not happen and the patient is imminently dying.

Many of the doctors interviewed for this study stated that their medical decision making generally would depend on patients’ wishes but as the empirical qualitative data shows, doctors did not discuss this in any detail with regard to the central theme of this paper; starting or stopping LMWH injections. It may appear incongruous that physicians would not talk about involving patients and their families in the decision making process for LMWH injections. However, it is clear that in this research this topic was not explicitly discussed by the doctors interviewed. This is not to state that doctors believe involving patients or families is unimportant or irrelevant but that the research team found no evidence either way to support or refute this notion.

The main limitation of this study is concerned with how representative the sample of self selecting doctors interviewed can ever be in a specialist area. Participants who agreed to an in depth interview may have been those who had a particular interest in advanced cancer patients and VTE – this may have been especially true of palliative medicine doctors and oncologists who see these patients on a frequent basis. Potential participants may have opted not to take part in the research if they felt their level of knowledge about cancer and VTE was not significant and that they may have incurred embarrassment as a result of this. This is a general problem in some areas of qualitative health research - and not specific as such - to our area of interest, but many of the participants were familiar with the principal investigators of the project via their clinical work.

The term "end of life" was deliberately not concretely defined during the interviews to allow for doctors to discuss their own decision making with reference to patients on this spectrum of advanced disease. We believe this approach has borne rich data about the ambiguity in clinical decision making for this patient group and we did not use a formal measure of performance status as that is not usual routine clinical practice in the UK across all settings.

There is little formal guidance or literature to help these clinicians with difficult decisions in the treatment of VTE for this group of patients. Whereas guidance on pain, agitation, nausea & vomiting, cardiopulmonary resuscitation, hydration & nutrition, ventilation and sedation at the end of life is available [16], no formal management guidelines exist for VTE specific to patients with advanced progressive cancer although Noble et al. have made recommendations based on the literature applied, where possible, to the palliative care population [5]. Although there is a large body of literature about end of life decision making, regarding a range of medical, consent and capacity issues, little has been written about the ethical decisions which doctors make regarding VTE in advanced cancer patients. No standardised practice or formal guidance is available to help doctors make these challenging and complicated decisions which is perhaps unsurprising given the highly individualistic and contextual nature of VTE treatment at the end of life.

## Conclusions

From the findings of this study, we can see how decisions are difficult for doctors to make for advanced cancer patients towards the end of life with VTE. These decisions are nuanced and often placed on a shifting continuum which takes into account both explicit but also subtle factors. The concept of “appropriateness” represents major elements of the decision making process which doctors engage with regarding this patient group, with little in the way of guidance to help doctors. Opinion on whether an action is considered “appropriate” can be polarised, even for doctors at the same grade and within the same specialty. This leads to implications for patient care, autonomy and life expectancy. LMWH as symptom control sometimes complicates matters further and can be an ethical dilemma in itself. In this paper, we have showed that treatment for VTE in this patient group is intrinsically bound to the doctors own moral and ethical framework.

## Ethics approval

The study was approved by Leeds East Research Ethics Committee on 17^th^ March 2010 (Ref: 10/H1306/13).

## Competing interests

SN, AM and MJ are co-directors of the TRAD Alliance. The TRAD Alliance is supported by an unrestricted educational grant from Pfizer. SN has given lectures on behalf of Pfizer, Sanofi Aventis, Leo Pharma and Boeringer Ingelheim; all fees are donated directly to charity. SN has also received research grant funding from Pfizer. AM is an advisory board member for Leo and for Pfizer and has received research grant funding from Pharmacia/Pfizer. There are no conflicts of interests declared from DD, LS, HP, and IW.

## Authors’ contributions

MJ & DD conceptualised the study idea and are both chief investigators for the study. MJ, DD, SN, IW & AM designed the study and obtained funding. LS & HP collected all the data. LS, HP & DD analysed data. LS interpreted all data and wrote the first draft of the manuscript. All authors reviewed and revised drafts of the manuscript and all authors approved the final version of the submitted manuscript.

## Funding

The study on which this paper is based was funded by the National Institute for Health Research, Research for Patient Benefit funding stream 2008 [PB-PG-1207-15033]. The funder had no role in the design, data collection, analysis or interpretation, write up or decision to submit the article for publication.

## Pre-publication history

The pre-publication history for this paper can be accessed here:

http://www.biomedcentral.com/1472-6939/13/22/prepub
